# Supplementary material for: Biological evaluation of a mechanical ventilator that operates by controlling an automated manual resuscitator. A descriptive study in swine
Source: PLoS One. 2022 Mar 3;17(3):e0264774. doi: 10.1371/journal.pone.0264774 (PMC8893637; doi:10.1371/journal.pone.0264774)
Supplement: S1 File — (PDF) [file pone.0264774.s001.pdf]

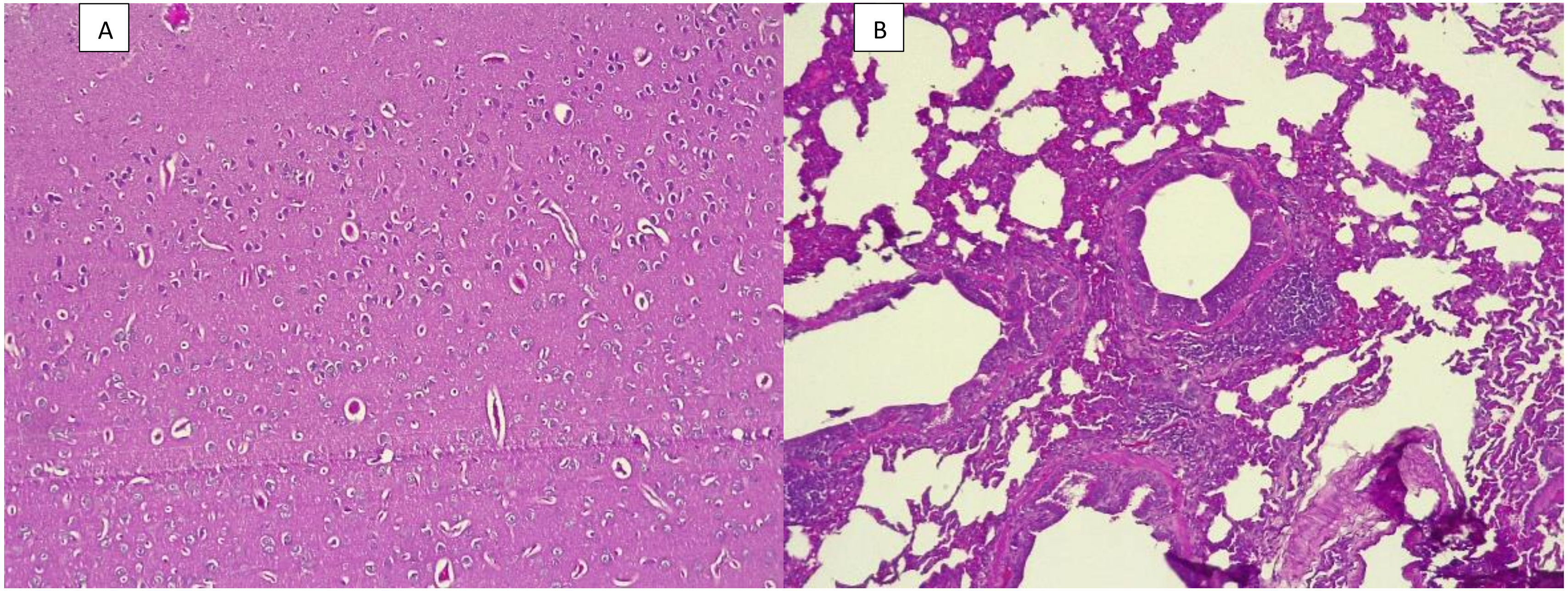

S1 Figure. Representative histopathology images. Representative pictures of (A) brain and (B) lung tissues. There were not changes associated with hypoxia; brain tissue shows only mild post-mortem retraction. Lung tissue shows mild lymphocyte interstitial infiltration probably related to previous pulmonary infection.

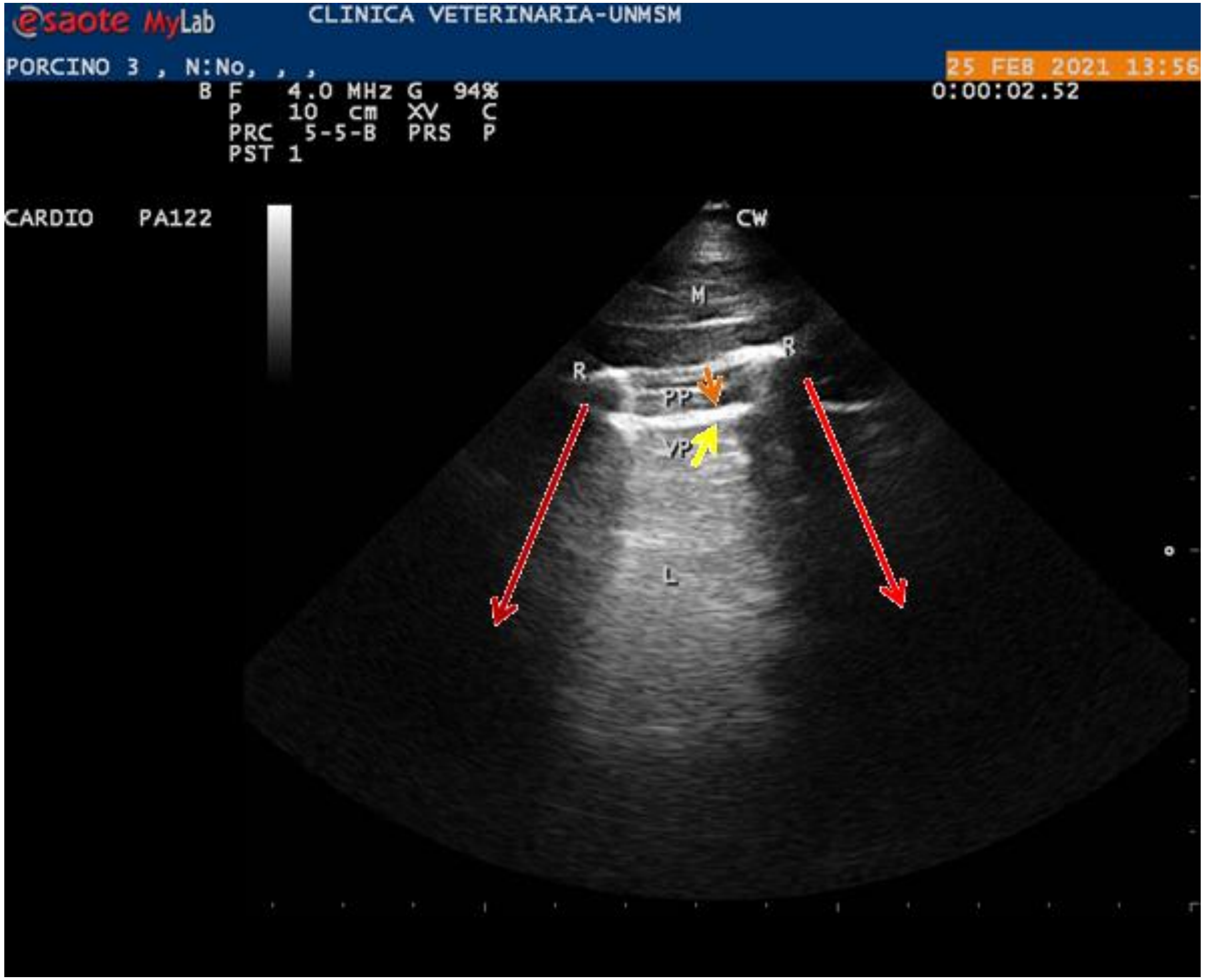

S2 Figure. Representative image of chest ultrasound. CW: Chest Wall; M: Muscle; PP: Parietal Pleura (orange arrow); VP: Visceral Pleura (yellow arrow); L: Lung; R: Ribs (red arrows).

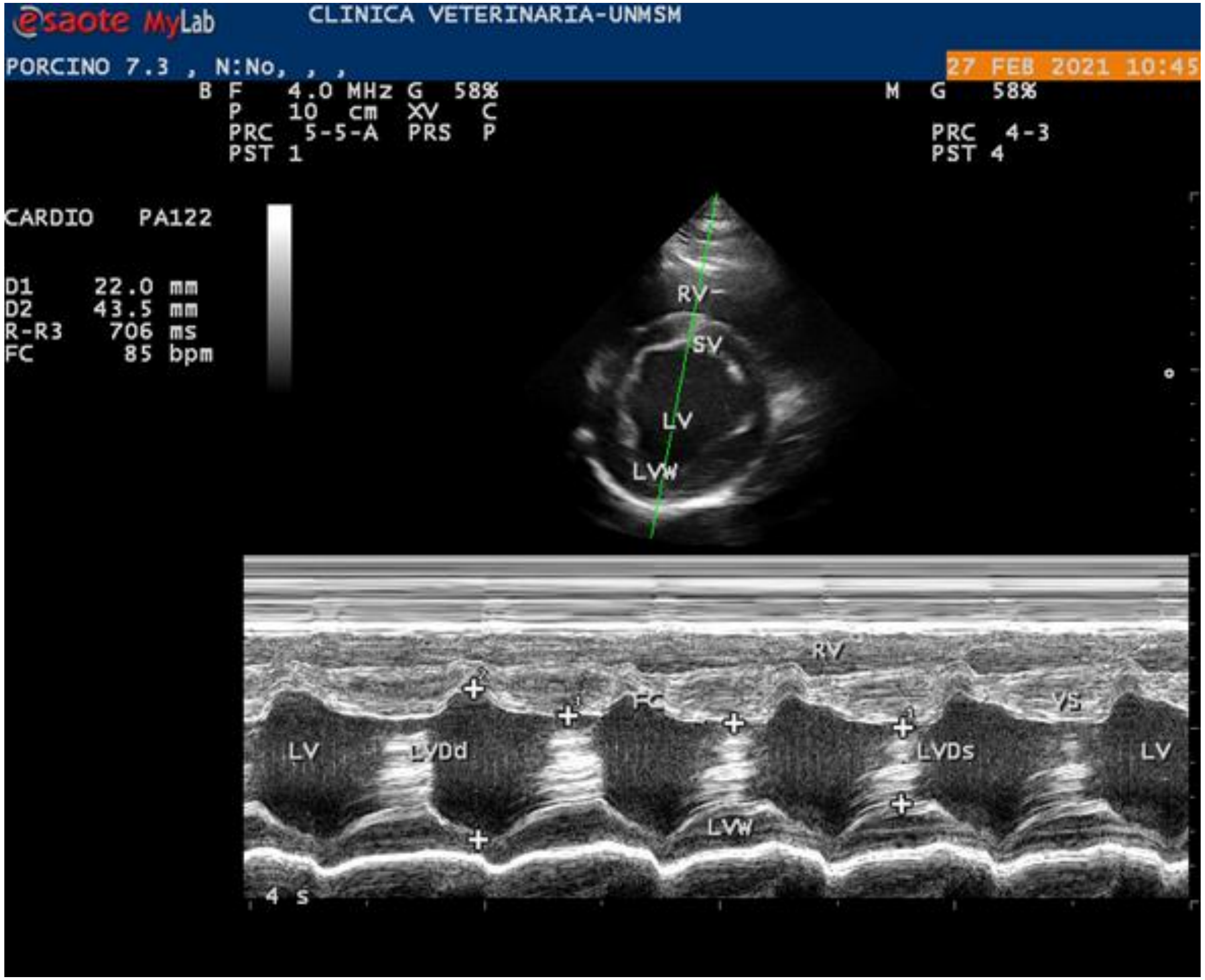

S3 Figure. Representative echocardiography image. Representative image describing contractile functions. RV: Right Ventricle; VS: Ventricular Septum; LV: Left Ventricle; LVW: Left Ventricular Wall.

S1 Table. Histopathological analysis per organ.

Swine #	Lung	Kidney	Liver	Brain	Heart	General comments
1	Right lung presents mild acute diffuse nonsuppurative interstitial pneumonia with mild BALT aggregate hyperplasia.	Nephrosis	Hepatic steatosis with centrilobular congestion.	No major changes.	No major changes.	The observed alterations suggest an anterior pneumonic process, no early hypoxic lesions are observed.
2	Severe acute diffuse nonsuppurative interstitial pneumonia in some areas, little peribronchial lymphocytic infiltration, in some bronchioles presence of exudate.	Mild acute multifocal nonsuppurative nephritis, nephrosis.	Hepatosis with few perilobular lymphocytic infiltration	Mild acute multifocal non-suppurative meningitis, gliosis, satellitosis, perivascular and neuronal edema, little neuronal death.	No major changes.	The observed alterations suggest an infectious process, no early hypoxic lesions are observed.
3	Moderate acute diffuse nonsuppurative interstitial pneumonia with BALT aggregation hyperplasia in some areas, scant infiltration of peribronchial lymphocytes, in some bronchioles presence of exudate.	Mild acute multifocal nonsuppurative nephritis, nephrosis.	Hepatosis	Mild acute focal non-suppurative meningitis with little blood extravasation, gliosis, satellitosis, perineuronal and vascular edema.	Hypertrophy of cardiac muscle.	The observed alterations suggest an infectious process, no early hypoxic lesions are observed.
4	Mild acute diffuse nonsuppurative interstitial pneumonia, scant number of lymphocytes around the bronchiole and bronchus.	Mild to moderate acute multifocal nonsuppurative nephritis with nephrosis.	Mild acute focal nonsuppurative hepatitis with hepatosis.	Few perivascular lymphocytes with perivascular edema.	No major changes.	The observed alterations suggest an infectious process (viral type?), No early hypoxic lesions are observed.
5	Severe acute diffuse nonsuppurative interstitial pneumonia in one of the lungs. The other presents mild acute diffuse nonsuppurative interstitial pneumonia.	Moderate acute multifocal nephritis with marrow atrophy and nephrosis.	No major changes.	No major changes.	Few lymphocytes in the interstitium.	The observed alterations suggest an infectious process (viral type?), No early hypoxic lesions are observed.

6	Moderate acute diffuse nonsuppurative interstitial pneumonia, with mild BALT aggregate hyperplasia.	Nephrosis, few lymphocytes in the interstitium.	Some hepatocytes with steatosis	Mild acute multifocal nonsuppurative meningoencephalitis with vacuolization in the area, satellitosis and gliosis.	No major changes.	The observed alterations suggest an infectious process (viral type?), No early hypoxic lesions are observed.
7	Moderate to mild acute diffuse nonsuppurative interstitial pneumonia, with mild BALT aggregate hyperplasia.	Nephrosis	Hepatosis	Few lymphocytes in the meninges with perivascular edema, satellitosis and gliosis.	No major changes.	The observed alterations suggest an infectious process, no early hypoxic lesions are observed.
8	Moderate to mild acute diffuse nonsuppurative interstitial pneumonia, with BALT aggregate hyperplasia.	Mild acute multifocal nonsuppurative interstitial nephritis with nephrosis.	No major changes.	Mild acute focal nonsuppurative encephalitis with vacuolization of the parenchyma, perivascular edema, satellitosis and gliosis. Few lymphocytes in the meninges.	No major changes.	The observed alterations suggest a previous infectious process, perhaps from a viral source. No early hypoxic lesions are observed.

Summary of histopathological findings in five organs: Lung, kidney, liver, brain, and heart analyzed for early lesions related to barotrauma or hypoxia. Results were reported by the Laboratory of Histology, Embryology and Animal Pathology at Faculty of Veterinary Medicine, Universidad Nacional Mayor de San Marcos. BALT: Bronchus-associated lymphoid tissue.

S2 Table. Comparison of baseline physiological values (t_0) and reference values.

	Current study		Reported in literature		
**Parameters**	**n**	**Mean**	**n**	**Mean**	**T-test p-value**
**Temperature (°C)**	8	36.6	30	38.9	0.157
**pH**	8	7.395	86	7.414	0.261
**HCO_3^- (mmHg)**	8	32.3	38	23.7	0.001
**Lactate (mmol/l)**	8	1.41	25	1.46	0.822
**BEecf (mmol/l)**	8	7.2	38	0.06	0.003
**SaO_2 (%)**	8	100	56	98.2	<0.0001
**PaO_2 (mmHg)**	8	334	86	161.9	<0.0001
**$PaCO_2$ (mmHg)**	8	52.9	86	39.5	0.218
**Respiratory rate (breaths/min)**	8	37.1	30	31	0.266
**Heart rate (beats/min)**	8	85.4	53	114	0.752
**Cardiac output (L/min)**	8	5.892	23	5.542	0.146

T-test was performed to analyze differences between physiological values described previously in the literature (Table 1) and our baseline data set (Table 3), significant differences were established at p-value <0.05.

S3 Table. Biochemical parameters.

Swine #	Time	pH	$PaCO_2$ (mmHg)	PaO_2 (mmHg)	BEecf (mmol/l)	HCO_3^- (mmHg)	$ETCO_2$ (mmHg)	SaO_2 (%)	Lactate (mmol/l)	Respiratory mechanism
1	t_0	7.329	61.4	361	6	32.3	34.0	100	1.27	Autonomous
2	t_0	7.428	45.1	327	5	29.8	31.0	100	1.88	Autonomous
3	t_0	7.372	52.9	289	5	30.7	32.0	100	1.28	Autonomous
4	t_0	7.412	48.0	317	6	30.6	32.0	100	1.81	Autonomous
5	t_0	7.363	60.0	529	9	34.2	36.0	100	0.63	Autonomous
6	t_0	7.407	55.2	414	10	34.8	36.0	100	1.20	Autonomous
7	t_0	7.431	48.6	267	8	32.3	34.0	100	1.44	Autonomous
8	t_0	7.415	52.3	169	9	33.5	35.0	100	1.80	Autonomous
1	t_1	7.402	47.0	240	4	29.2	31.0	100	2.57	Mechanical
2	t_1	7.312	61.1	282	5	30.9	33.0	100	4.32	Mechanical
3	t_1	7.290	67.4	29	6	32.4	34.0	45	0.88	Mechanical
4	t_1	7.267	68.7	86	4	31.3	33.0	94	1.46	Mechanical
5	t_1	7.371	53.0	120	5	30.7	32.0	98	0.59	Mechanical
6	t_1	7.362	52.3	53	4	29.7	31.0	85	0.75	Mechanical
7	t_1	7.467	43.7	179	8	31.6	33.0	100	1.71	Mechanical
8	t_1	7.418	46.1	149	5	29.8	31.0	99	3.78	Mechanical

1	t_2	7.389	52.0	248	6	31.4	33.0	100	1.83	Mechanical
2	t_2	7.369	48.2	278	3	27.8	29.0	100	6.95	Mechanical
3	t_2	7.332	56.7	104	4	30.1	32.0	97	1.90	Mechanical
4	t_2	7.398	50.2	197	6	30.9	32.0	100	1.21	Mechanical
5	t_2	7.525	36.5	135	7	30.2	31.0	99	0.57	Mechanical
6	t_2	7.522	37.9	213	8	31.1	32.0	100	1.35	Mechanical
7	t_2	7.509	40.1	158	9	31.9	33.0	100	1.44	Mechanical
8	t_2	7.429	45.9	169	6	30.4	32.0	100	4.30	Mechanical
1	t_3	7.439	44.3	247	6	30.0	31.0	100	1.53	Mechanical
2	t_3	7.364	48.4	279	2	27.6	29.0	100	7.83	Mechanical
3	t_3	7.369	53.3	148	5	30.7	32.0	99	1.63	Mechanical
4	t_3	7.416	52.6	219	9	33.8	35.0	100	1.05	Mechanical
5	t_3	7.490	34.6	142	3	26.4	27.0	99	3.09	Mechanical
6	t_3	7.557	35.5	207	9	31.6	33.0	100	1.18	Mechanical
7	t_3	7.527	39.2	186	10	32.5	34.0	100	1.28	Mechanical
8	t_3	7.499	40.8	204	9	31.7	33.0	100	2.96	Mechanical
1	t_4	7.556	35.3	285	9	31.3	32.0	100	1.84	Mechanical
2	t_4	7.362	44.7	267	0	25.3	27.0	100	9.21	Mechanical
3	t_4	7.422	48.1	127	7	31.3	33.0	99	0.98	Mechanical

4	t_4	7.444	44.9	222	7	30.8	30.8	100	1.18	Mechanical
5	t_4	7.470	36.4	142	3	26.5	28.0	99	4.74	Mechanical
6	t_4	7.483	43.3	226	9	32.4	34.0	100	1.16	Mechanical
7	t_4	7.537	40.0	170	11	34.0	35.0	100	1.45	Mechanical
8	t_4	7.532	38.7	208	10	32.5	34.0	100	1.45	Mechanical
1	t_5	7.460	44.5	277	8	31.6	33.0	100	1.06	Mechanical
2	t_5	7.355	48.2	290	1	26.9	28.0	100	8.89	Mechanical
3	t_5	7.482	42.7	194	8	31.9	33.0	100	1.07	Mechanical
4	t_5	7.434	48.7	280	8	32.6	32.0	100	1.02	Mechanical
5	t_5	7.416	45.7	252	5	29.4	31.0	100	4.57	Mechanical
6	t_5	7.475	43.3	235	8	31.9	33.0	100	1.14	Mechanical
7	t_5	7.515	42.1	212	11	34.0	35.0	100	1.44	Mechanical
8	t_5	7.511	42.2	185	11	33.8	35.0	100	1.34	Mechanical
1	t_6	7.472	42.6	277	8	31.1	32.0	100	0.81	Mechanical
2	t_6	7.193	72.4	69	0	27.9	30.0	88	7.94	Mechanical
3	t_6	7.478	42.9	194	8	31.8	33.0	100	0.99	Mechanical
4	t_6	7.492	43.2	230	10	33.1	34.0	100	0.94	Mechanical
5	t_6	7.474	41.6	202	7	30.6	32.0	100	1.98	Mechanical
6	t_6	7.444	47.2	211	8	32.3	34.0	100	1.00	Mechanical

7	t_6	7.500	42.7	178	10	33.3	35.0	100	1.47	Mechanical
8	t_6	7.486	45.8	177	11	34.6	34.6	100	1.14	Mechanical
1	t_7	7.520	40.1	249	10	32.7	34.0	100	0.69	Mechanical
2	t_7	7.371	54.8	178	6	31.7	33.0	100	3.79	Mechanical
3	t_7	7.461	44.7	124	8	31.9	33.0	99	0.92	Mechanical
4	t_7	7.513	40.3	155	9	32.4	34.0	100	0.87	Mechanical
5	t_7	7.473	42.2	153	7	30.9	32.0	99	1.39	Mechanical
6	t_7	7.420	51.5	278	9	33.4	35.0	100	0.90	Mechanical
7	t_7	7.464	48.3	159	11	34.6	36.0	99	1.28	Mechanical
8	t_7	7.503	44.0	204	11	34.6	34.6	100	1.16	Mechanical

Values of different parameters of arterial blood biochemistry obtained at the different time-points of the pre-clinical trial as explained in Figure 2. pH: potential of hydrogen, HCO_3^-: bicarbonate ion, BEecf: base excess in the extracellular fluid compartment, SaO_2: arterial oxygen saturation, PaO_2: arterial oxygen pressure, $PaCO_2$: arterial carbon dioxide pressure, $ETCO_2$: end-tidal carbon dioxide.

S4 Table. Cardio-respiratory parameters at different time-points.

Swine #	Time	Respiratory rate (breaths/min)	Heart rate (beats/min)	LVEDV (mL)	LVESV (mL)	Cardiac output (L/min)
1	t_0	40	83	nr	nr	3.203
2	t_0	56	101	nr	nr	8.171
3	t_0	40	81	nr	nr	6.460
4	t_0	48	69	nr	nr	3.969
4	t_4	nr	69	70.42	12.89	3.969
4	t_6	nr	60	105.44	21.01	5.065
5	t_0	32	70	105.44	14.76	6.347
5	t_4	nr	105	75.95	8.39	7.093
5	t_6	nr	86	79.9	20.37	5.120
6	t_0	25	86	83.07	18.72	5.534
6	t_4	nr	77	95.36	25.55	5.375
6	t_6	nr	70	73.79	10.14	4.455
7	t_0	20	84	105.44	22.09	7.001
7	t_4	nr	74	95.41	16.20	5.713
7	t_6	nr	87	87.68	12.25	6.562
8	t_0	36	99	67.53	17.34	4.968

8	t_4	nr	92	82.61	12.89	6.414
8	t_6	nr	84	85.36	23.68	5.181

Values of cardiovascular and respiratory parameters measured at t_0, t_4 (2 hours) and t_6 (4 hours) of the pre-clinical trial as explained in Figure 2. LVEDV: Left ventricular end diastolic volumen; LVESV: left ventricular end systolic volumen; nr: not recorded.
