# Supplementary material for: Biological evaluation of a mechanical ventilator that operates by controlling an automated manual resuscitator. A descriptive study in swine
Source: PLoS One. 2022 Mar 3;17(3):e0264774. doi: 10.1371/journal.pone.0264774 (PMC8893637; doi:10.1371/journal.pone.0264774)
Supplement: Attachment — Submitted filename: Manuscript number PONE-D-21-30195.docx [file pone.0264774.s002.docx]

The manuscript entitled “Biological evaluation of a mechanical ventilator that operates by controlling an automated manual resuscitator. A descriptive study in swine” ( Manuscript number PONE-D-21-30195) presented the characteristics of a new ventilator device.

I congratulate the authors for the study, risen from the need to dispose of larger amount of mechanical ventilation during COVID- 19 pandemics; this reason makes this study relevant.

However, there are several points that needs to be addressed in order to make this paper worthy of publication.

The characteristic of the tested ventilator highlighted in the title (“…that operates by controlling an automated manual resuscitator”) is not considered at all into the manuscript.

Introduction. In my opinion, retracing the evolution of the COVID- 19 pandemics (i.e. Specifying date of the first case in China and in Perù) is not pertaining to the aim of the study. In order to put the new mechanical ventilator production into the context of the COVID – 19 pandemics, the authors should instead provide more precise epidemiologic data (es. number of patients admitted, patients requiring mechanical ventilation).

From line 76 to 79. Please provide some details about the so called “operating mechanism” of the Masi ventilator. In this context, mentioning the commercial name of other similar ventilator may be confounding and not relevant. Which is the meaning of “less complex mechanical ventilator systems”? In other words, please brefly describe the differences between the Masi ventilator and others, commonly used in the ICU.

From line 85 to 87. Please rephrase this sentence.

From line 96 to 98.The aims of the study should be precisely defined at this point, together with the primary endpoints.

Methods. The experimental design should be clearer then it is. Moreover, sample size calculation should be explicated for each proposed study included in the experimental design.

Line 216-217. Variables measured by echocardiography should be defined and subsequently described into the results section. The assessment “there were no alterations in the functioning of these organs that could indicate injuries related to the use of Masi mechanical ventilator” (line348-349) is a qualitative evaluation.

Line 251. Pigs (at baseline) are not patients.

Table 3. Reference values showed in a separated figure are unclear.
